# Gut dysbiosis promotes prostate cancer progression and docetaxel resistance via activating NF-κB-IL6-STAT3 axis

**DOI:** 10.1186/s40168-022-01289-w

**Published:** 2022-06-16

**Authors:** Weibo Zhong, Kaihui Wu, Zining Long, Xumin Zhou, Chuanfan Zhong, Shuo Wang, Houhua Lai, Yufei Guo, Daojun Lv, Jianming Lu, Xiangming Mao

**Affiliations:** 1 Department of Urology, Zhujiang Hospital, Southern Medical University, Guangzhou, 510280 China; 2 Department of Urology, The Third Affiliated Hospital of Guangzhou Medical University, Guangzhou, 510150 China; 3 Department of Andrology, Guangzhou First People’s Hospital, School of Medicine, Guangzhou Medical University, Guangzhou, 510180 China

**Keywords:** Gut dysbiosis, Prostate cancer, *Proteobacteria*, Lipopolysaccharide, NF-κB-IL6-STAT3 axis

## Abstract

**Background:**

The gut microbiota is reportedly involved in the progression and chemoresistance of various human malignancies. However, the underlying mechanisms behind how it exerts some effect on prostate cancer, as an extra-intestinal tumor, in a contact-independent way remain elusive and deserve exploration. Antibiotic exposure, one of the various factors affecting the gut microbiota community and capable of causing gut dysbiosis, is associated with multiple disorders. This study aims to preliminarily clarify the link between gut dysbiosis and prostate cancer.

**Results:**

First, we discovered that perturbing the gut microbiota by consuming broad-spectrum antibiotics in water promoted the growth of subcutaneous and orthotopic tumors in mice. Fecal microbiota transplantation could transmit the effect of antibiotic exposure on tumor growth. Then, 16S rRNA sequencing for mouse feces indicated that the relative abundance of *Proteobacteria* was significantly higher after antibiotic exposure. Meanwhile, intratumoral lipopolysaccharide (LPS) profoundly increased under the elevation of gut permeability. Both in vivo and in vitro experiments revealed that the NF-κB-IL6-STAT3 axis activated by intratumoral LPS facilitated prostate cancer proliferation and docetaxel chemoresistance. Finally, 16S rRNA sequencing of patients’ fecal samples revealed that *Proteobacteria* was enriched in patients with metastatic prostate cancer and was positively correlated with plasma IL6 level, regional lymph node metastasis status, and distant metastasis status. The receiver operating characteristic (ROC) curves showed that the relative abundance of *Proteobacteria* had better performance than the prostate-specific antigen (PSA) level in predicting the probability of distant metastasis in prostate cancer (area under the ROC curve, 0.860; *p* < 0.001).

**Conclusion:**

Collectively, this research demonstrated that gut dysbiosis, characterized by the enrichment of *Proteobacteria* due to antibiotic exposure, resulted in the elevation of gut permeability and intratumoral LPS, promoting the development of prostate cancer via the NF-κB-IL6-STAT3 axis in mice. Considering findings from human patients, *Proteobacteria* might act as an intestinal biomarker for progressive prostate cancer.

Video Abstract

**Supplementary Information:**

The online version contains supplementary material available at 10.1186/s40168-022-01289-w.

## Introduction

The statistics from GLOBOCAN 2020 estimated that the incidence of prostate cancer was the second highest, while its mortality rate ranked fifth, among men worldwide [1]. In the USA, new cases of prostate cancer have surpassed lung cancer to occupy the first place, while the mortality rate ranks second, showing an upward trend in recent years and presenting a huge challenge to the healthcare system [2]. Therefore, it is necessary to elucidate the mechanisms for prostate cancer progression and to develop novel therapeutic methods.

In the past decade, an increasing number of studies have focused on the role of gut microbiota in tumor progression and treatment [3–5]. However, most available studies highlighted the connection between gut microbiota and tumors that make contact with microorganisms directly, and little is known regarding how gut microbes affect extra-intestinal malignancies, such as prostate cancer. Recently, Pernigoni found that commensal bacteria promote endocrine resistance in prostate cancer through androgen biosynthesis [6], while Daisley showed that the gut commensal *Akkermansia muciniphila* affected the biotransformation of abiraterone acetate in patients with castration-resistant prostate cancer (CRPC) [7]. It has also been reported that short-chain fatty acids (SCFAs) derived from the gut microbiota may promote prostate cancer growth via the insulin growth factor-1 signaling pathway [8].

Diet, drugs, disease status, and antibiotics are known to alter the composition of gut microbiota. Antibiotics, developed to work against pathogens initially, have attracted more attention due to the collateral damage caused to the gut microbiota, causing a variety of immunological, inflammatory, and metabolic disorders [9–11]. One study showed that greater accumulative exposure to systemic antibiotic usage, particularly with broad-spectrum microbial coverage, may be correlated to a higher incidence of inflammatory bowel disease (IBD) [12]. Furthermore, a series of studies have linked antibiotic usage to an increased risk of colorectal cancer, weakened effect of immune checkpoint inhibitor (ICI) on bladder cancer, and lower survival for patients with advanced hepatocellular carcinoma during treatment with ICI [13–15]. In spite of these observations and the link between the gut microbiota and prostate cancer described above, it remains an unsolved mystery whether and how antibiotic exposure affects tumor progression or impairs therapeutic effectiveness in prostate cancer by altering the composition of gut microbiota.

Available reviews have concluded that microbiome contributes to tumor development mainly through inflammation, immunity, metabolites, and antigen simulation [16, 17]. Inflammation has been shown to accelerate the progression of various tumors, including prostate cancer [18]. The mechanisms involved are oxidative stress, epigenetic changes, and shaping of the inflammatory tumor microenvironment [19].

In this study, we hypothesized that as an inflammatory trigger factor, gut dysbiosis caused by antibiotic exposure activates the inflammatory signaling pathway and contributes to prostate cancer progression. To verify this assumption, a gut dysbiosis murine model was established by oral intake of broad-spectrum antibiotics, and 16S rRNA sequencing was applied to determine the composition of the gut microbiota. We found that antibiotic exposure effectively promoted prostate cancer growth, which was mediated by the gut microbiota. The relative abundance of *Proteobacteria* was higher after antibiotic exposure, and intratumoral lipopolysaccharide (LPS) was increased under the elevation of gut permeability, playing a critical role in prostate cancer progression via the NF-κB-IL6-STAT3 axis.

## Materials and methods

### Reagents and antibodies

Reagents: ampicillin (A8180-2, Solarbio, China); colistin (R002831, Rhawn, China); neomycin and vancomycin (N814740 and V820413, Macklin, China); LPS (L8880, Solarbio, China); BAY-11-7082 (S1523, Beyotime, China); Stattic (A2224, APExBIO, USA); Docetaxel (A4394, APExBIO, USA); DMSO (D8370, Solarbio, China); PEG300 (IP9020, Solarbio, China); Tween-80 (T8360, Solarbio, China); DAPI (C0065, Solarbio, China); DAB (ZLI-9018, ZSGB-BIO, China).

Antibodies: Ki-67 (ab15580, abcam, UK); LPS (MAB526Ge22, Cloud-Clone, China); p65 (AF1234, Beyotime, China); p-p65 (Ser536) (AF2006, Affinity, USA); p-STAT3 (Tyr705) (AF3293, Affinity, USA); STAT3 (AF1492, Beyotime, China); Antibody-IL6 (AF-406-SP, R&D, USA); C-myc (AF0358, Affinity, USA); Cyclin D1 (AF0931, Affinity, USA); Bcl-2 (AF6285, Beyotime, China); Survivin (AF6017, Affinity, USA); β-actin (AF5003, Beyotime, China); β-tubulin (AF1216, Beyotime, China). Second antibodies: HRP-conjugated goat anti-rabbit IgG (cw0103s, CWBIO, China); HRP-conjugated goat anti-mouse IgG (cw0102s, CWBIO, China); fluorescent Cy3-conjugated goat anti-rabbit IgG (BA1032, BOSTER, China).

### Animal models

The animals used in this study were C57BL/6J male mice (SPF level) aged 6–8 weeks old. All animal experiments were approved by the Ethics Committee for Animal Care and Research at Zhujiang Hospital, Southern Medical University.

All the mice were fed ordinary food. Drinking water was added with broad-spectrum antibiotics for the Abx group and without for the NC group. The broad-spectrum antibiotic formula includes ampicillin, colistin, neomycin, and vancomycin. The used dosage of ampicillin, colistin, neomycin, and vancomycin is 0.25g, 0.25g, 1.25g, and 0.0625g, respectively, and all of them were dissolved in a total of 250 mL ddH20. Subcutaneous and orthotopical tumors were established with a murine prostate cancer cell line RM-1 (10^7^ cells/mL) after mice received antibiotic pretreatment for 7–10 days. The injection volume for subcutaneous tumors was 100 μL/mouse and 50 μL/mouse for orthotopic tumors. All procedures were performed while the mice were under anesthesia (12.5% tribromoethanol, 0.25 mL/10 g of body weight by intraperitoneal injection).

Subcutaneous tumors were inoculated on the right lower back, near the right thigh. The procedure for orthotopical tumor transplantation was as follows: a transverse incision of 1 cm was made in the lower abdomen to expose the bladder (a transparent globular organ). Tweezers were used to lift the bladder, revealing the prostate below it. A 29-gauge needle was gently inserted into the prostate; then, the cell suspension was injected. The procedure would be deemed successful if the prostate bulged without leakage. Finally, the anatomical positioning of each organ was restored, and the incision was sutured.

For fecal microbiota transplantation (FMT), mice drank antibiotic-contained water for 3 days to reduce the intestinal bacterial load and then switched to ordinary water. Fresh excreted feces from the mice in the Abx or NC group were collected, and 0.3 g of feces was added to 2–3 mL of sterile water; any solid impurities were removed using a 70-μm nylon filter sieve after homogenization. The fecal bacterial suspension was prepared and injected orally by a gavage needle at 200 μL/mouse/day.

For drug treatment, Stattic powder (2.5 mg/kg body weight) was dissolved in 5% DMSO, 30% PEG300, 5% Tween-80, and 60% ddH20, and docetaxel powder (10 mg/kg body weight) was dissolved in 5% DMSO, 40% PEG300, 5% Tween-80, and 50% ddH20. When the subcutaneous tumor volume was approximately 100 mm^3^, drugs were intra-peritoneally injected into mice once every 2 days, 4–5 times in total. The tumor volume measurement formula is as follows: volume = (length × width^2^)/2.

The feeding period was 4–6 weeks. Samples of tumor, feces, blood, and intestine were collected.

### Clinical patients

The collection of clinical patient samples and information was approved by the Clinical Research Center and Ethics Committee at Zhujiang Hospital, Southern Medical University. Patients with prostatic diseases (benign prostatic hyperplasia or prostate cancer) admitted to the inpatient Department of Urology of Zhujiang Hospital from January 2019 to January 2021 were included. Fecal samples and clinical parameters, such as age, body mass index (BMI), blood index (PSA, IL6), and tumor TNM stage, were collected.

The study inclusion criteria were as follows: (1) men aged 55–90 years and (2) men admitted to the hospital with benign prostatic hyperplasia or prostate cancer. Exclusion criteria for the study included (1) severe neuropathy, mental illness, or severe heart and lung disease; (2) a history of taking antibiotics or probiotics within the previous 3 months; and (3) gastrointestinal disease.

A total of 35 patients were finally recruited. During hospitalization, fecal samples were collected before any treatment or prostate biopsy by using a sterile specimen bag to facilitate patient fecal sample collection. Sterile forceps were used to separate feces into a 1.5-mL cryopreservation tube, which was quick-frozen with liquid nitrogen within 30 min and refrigerated at −80 °C.

### Cell culture

The murine prostate cancer cell line RM-1 was obtained from Procell Biotechnology (Wuhan, China). The human prostate cancer cell line DU145 was obtained from the Cell Resources Center of the Shanghai Institutes for Biological Sciences (Chinese Academy of Sciences, Shanghai, China). RM-1 and DU-145 were cultured with Roswell Park Memorial Institute (RPMI) 1640 medium (containing 10% fetal bovine serum (FBS) with 1% penicillin and streptomycin) at 37°C and 5% CO_2_. The tumor cells were cultured with LPS (dissolved in RPMI 1640 medium) for 24 h. Then, the medium was removed, and the cells were cleaned three times with phosphate-buffered saline (PBS) and reintroduced into a fresh medium for another 24 h of cultivation. Subsequently, the medium was collected and mixed with the new medium at a ratio of 1:1 to form the conditional medium (CM).

### Immunohistochemistry and HE staining

The tumor and colon tissues were fixed with 4% paraformaldehyde and were cut into 4-μm-thick paraffin sections. Both in the immunohistochemistry and the HE staining procedures, paraffin sections were roasted, dewaxed, and hydrated first.

Immunohistochemistry: EDTA (pH = 8.0) was applied to antigen repair using the pressure cooker. The sections were incubated with the primary antibody at 4°C overnight after being treated with 3% H_2_O_2_ for 10 min to inactivate endogenous peroxidase and blocked with 5% goat serum for 1 h. The primary antibodies include Ki-67 (1:100), LPS (1:200), p-p65 (1:250), p-STAT3 (1:250), C-myc (1:250), Cyclin D1 (1:250), Bcl-2 (1:250), and Survivin (1:250). The sections were incubated with the secondary antibody (1:500) for 1 h at room temperature the next day. Finally, DAB was used to detect the expression levels of targeted proteins, and nuclei were stained with hematoxylin. For statistical analysis, five different fields were taken from each section. Mean optical density (integral optical density/area) was used to evaluate the expression levels of LPS, and positive cell counting was applied for the rest. The analysis process was completed by ImageJ.

HE staining: Eosin was stained for cytoplasm and extracellular matrix. A histological score of colon tissues was conducted according to reference [20] and finished by two individuals independently.

### Immunofluorescence staining

The tumor cells were inoculated in 24-well plates and collected after a 24-h drug intervention. After the supernatant was removed, cells were cleaned with PBS, fixed with 4% paraformaldehyde for 20 min, permeated by 0.5% Triton X-100 at room temperature for 10 min, blocked by 5% FBS for 1 h, and inoculated with the primary antibodies, including p-p65 (1:250) and p-STAT3 (1:250), at 4°C overnight. On the second day, a fluorescent secondary antibody (1:500) was incubated at room temperature for 1 h. DAPI was used to stain nuclei. A fluorescent microscope photographed the samples at a 554-nm excitation wavelength.

### ELISA

The fecal suspension was prepared as described above, and LPS levels in feces were measured by the ELISA kit (Cloud-Clone, China).

The IL6 ELISA kit (KeyGEN, China) was used to detect IL6 levels in cell supernatant, tumor tissue lysate, and mouse serum. Tumor tissues of the same weight were mixed with the same volume of RIPA lysate and homogenized thoroughly by the grinding machine. The supernatant obtained after centrifugation was tumor tissue lysate.

After sacrificing the mice, we collected blood by cardiac puncture, placed them at room temperature for 1–2 h, and centrifuged them at 3000 rpm/min for 15 min. The upper fluid was absorbed to obtain serum, which was used to detect the levels of IL6 and LPS. All procedures were carried out according to the kit instructions.

### Western blot

The mixture of RIPA lysate, phosphatase inhibitors, and protease inhibitors was used to extract proteins from cells and tissues. All steps were followed as per standard procedures. The primary antibodies used (all at 1:1000) were LPS, p65, p-p65, p-STAT3, STAT3, C-myc, Cyclin D1, Bcl-2, Survivin, β-actin, and β-tubulin. The second antibodies used (all at 1:5000) were HRP-conjugated goat anti-rabbit IgG and HRP-conjugated goat anti-mouse IgG. The bands were visualized with ECL hypersensitive luminescence solution (WBKLS0100, Millipore, USA). The density of the band was calculated by Image Lab. Representative images of the western blot of cells from one of the three independent experiments are presented. β-tubulin and β-actin were used as reference genes.

### Real-time quantitative PCR

The TriZOL reagent (15596-026, Thermo Fisher Scientific, China) was applied to extract cell total RNA according to instructions. The isolated RNA was applied to synthesize cDNA with the PrimeScript RT reagent Kit (RR036A, Takara, China) according to protocols. The primers used in the study were obtained from PrimerbBank (https://pga.mgh.harvard.edu/primerbank/), were verified by Primer-Blast in NCBI, and were synthesized by TsingKe Biological Technology (Guangzhou, China). The primers’ sequences are shown in Table S1.

Real-time quantitative PCR was performed by SYBR Green PCR Master Mix (RR820A, Takara, China) and operated via ABI QuantStudio 3 (Applied Biosystems, USA). The experiments were repeated in triplicate.

Amplification process: 95°C for 80 s, 40 cycles for 95°C for 5 s, 60°C for 30 s, and 72°C for 30 s. The relative quantitative method (2^^-△△Ct^) was used to calculate the relative fold change of genes. β-actin was applied as the reference gene.

### LinkedOmics database

To produce the volcano plot and perform the GSEA analysis and Pearson correlation test (between p-STAT3 and p-p65), we used the LinkedOmics database (http://www.linkedomics.org/login.php) [21]. The samples used in this database are all prostate cancer tissue.

#### The volcano plot and GSEA analysis

Cancer type: prostate adenocarcinoma (PRAD); sample cohort: TCGA_PRAD; search data type: RNAseq; attribute: IL6; subset: gender, male (*n* = 497); target data type: RPPA; statistical method: Spearman correlation test. Enrichment method: GSEA; enrichment categories: KEGG pathway.

#### Pearson correlation test

Cancer type: prostate adenocarcinoma (PRAD); sample cohort: TCGA_PRAD; search data type: RPPA; attribute: STAT3|STAT3_pY705; subset: histological_type, prostate adenocarcinoma, acinartype (*N* = 484); target data type: RPPA; target gene: NFKB1|NF-kB-p65_pS536; statistical method: Pearson correlation test.

### Edu and TUNEL assays

Edu (5-ethynyl-2′-deoxyuridine) and TUNEL (terminal deoxynucleotidyl transferase-mediated dUTP-biotin nick-end labeling) assays detect cell proliferation and apoptosis activity. 5×10^3^~2×10^4^ cells/well were inoculated in 24-well plates. For the TUNEL assays, cells were cultured for 24 h under three conditions: blank, CM alone, and CM with Stattic. Following the above treatments, we added docetaxel to the media for the 24-h cultivation. For the Edu assays, cells were treated with CM or CM with Stattic for 24 h. All steps were carried out according to the kit instructions (Beyotime, China). The cell proliferation or apoptosis rate was shown by the ratio of Edu- or TUNEL-positive cells (stained with green fluorescence) to Hoechst- or DAPI-positive cells (stained with blue fluorescence). There were three biological replicates in these experiments.

### Cell viability and clone formation assays

For the cell viability assay, 5000 cells/well were inoculated in 96-well plates and treated with different conditions as described above, respectively. The optical density (OD) value was measured by a microplate reader at 450 nm after incubation with CCK8 reagent (K1018, APExBIO, USA) for 2–3 h. The experiment was repeated three times.

For the clone formation assay, 500–1000 cells/well were inoculated in 6-well plates and treated with different conditions for 14 days. Finally, clone communities stained by crystal violet were counted by ImageJ. There were three biological replicates in this experiment.

### 16S rRNA sequencing for fecal samples

All fecal samples were subjected to 16S rRNA sequencing. The general process is described briefly as follows:

- (i) DNA extraction: We used the HiPure Stool DNA Kits (Magen, Guangzhou, China) to extract microbial DNA according to the instructions.
- (ii) PCR amplification: We used specific primers with barcode (341F, 5′-CCTACGGGNGGCWGCAG-3′ and 806R, 5′-GGACTACHVGGGTATCTAAT-3′) to amplify the 16S rDNA target region (V3-V4). The amplification process is as follows: 95°C for 5 min, 95°C for 1 min with 30 cycles, 60°C for 1 min, and 72°C for 1 min, with a final extension of 7 min at 72°C. PCR reactions were performed in triplicate, and the reaction system consisted of 10 μL of 5× Q5@ Reaction Buffer, 10 μL of 5× Q5@ High GC Enhancer, 1.5 μL of 2.5 mM dNTPs, 1.5 μL of each primer (10 μM), 0.2 μL of Q5@ High-Fidelity DNA Polymerase, and 50 ng of template DNA, for a total of 50 μL.
- (iii) Amplicon purification, quantification, and sequencing: Amplification products were purified by the AxyPrep DNA Gel Extraction Kit (Axygen Biosciences, Union City, CA, USA) according to the instructions and quantified by the ABI StepOnePlus Real-Time PCR System (Life Technologies, Foster City, USA). Purified products were pooled in equimolar and paired-end sequenced (PE250) on an Illumina platform according to protocols.
- (iiii) Quality control and clustering: Raw data containing more than 10% unknown nucleotides (N) and less than 50% bases with a quality (*Q*-value) greater than 20 was further filtered using FASTP [22] (version 0.18.0). Paired-end clean reads were merged as raw tags by FLSAH [23] (version 1.2.11) with a minimum overlap of 10 bp and mismatch error rates of 2%. Noisy sequencing of raw tags was filtered under special filtering criteria [24] to obtain the high-quality clean tags. The filtering criteria are as follows: (1) break raw tags from the first low-quality base site where the number of bases in the continuous low-quality value (the default quality threshold is ≤3) reaches the set length (the default length is 3 bp); (2) filter tags whose continuous high-quality base length is less than 75% of the tag length. The clean tags were clustered into operational taxonomic units (OTUs) of ≥97% similarity by the UPARSE [25] (version 9.2.64) pipeline. The UCHIME algorithm [26] was applied to delete all chimeric tags, and finally, effective tags were acquired for further analysis. The most abundant tag sequencing was chosen as the representative sequencing in each cluster.
- (iiiii) Taxonomy annotation: with a confidence threshold of 0.8, the representative OTU sequencings were classified into organisms using a naive Bayesian model via RDP classifier [27] (version 2.2) based on the SILVA database [28] (version 132).

Raw data has been deposited in the NCBI Sequence Read Archive (SRA) database and is now available (accession number: PRJNA 792067).

All procedures were performed by Gene Denovo Biotechnology (Guangzhou, China). Bioinformatic analysis was performed via Omicsmart, a Dynamic Real-Time Interactive Online Platform for Data Analysis (http://www.omicsmart.com).

### Statistical analysis

The data presented is mean ± SEM. For statistical analysis, the following software was used: GraphPad Prism 8, Origin 2021, MedCalc Version 20, and IBM SPSS Statistics Version 22. Partial results were derived from Omicsmart. An unpaired Student’s *T*-test was used to analyze continuous data from two groups, and data from three or more groups was tested using one-way ANOVA or Kruskal-Wallis, as appropriate. The chi-square test was used to analyze categorical data. The Pearson or Spearman test was used for correlation analysis as appropriate. There was a significant difference when *p* < 0.05. All *p* values were two-tailed.

## Results

### Drinking water containing broad-spectrum antibiotics promoted prostate cancer growth, which was associated with disturbing the gut microbiota in C57BL/6J mice

**Fig. 1:**
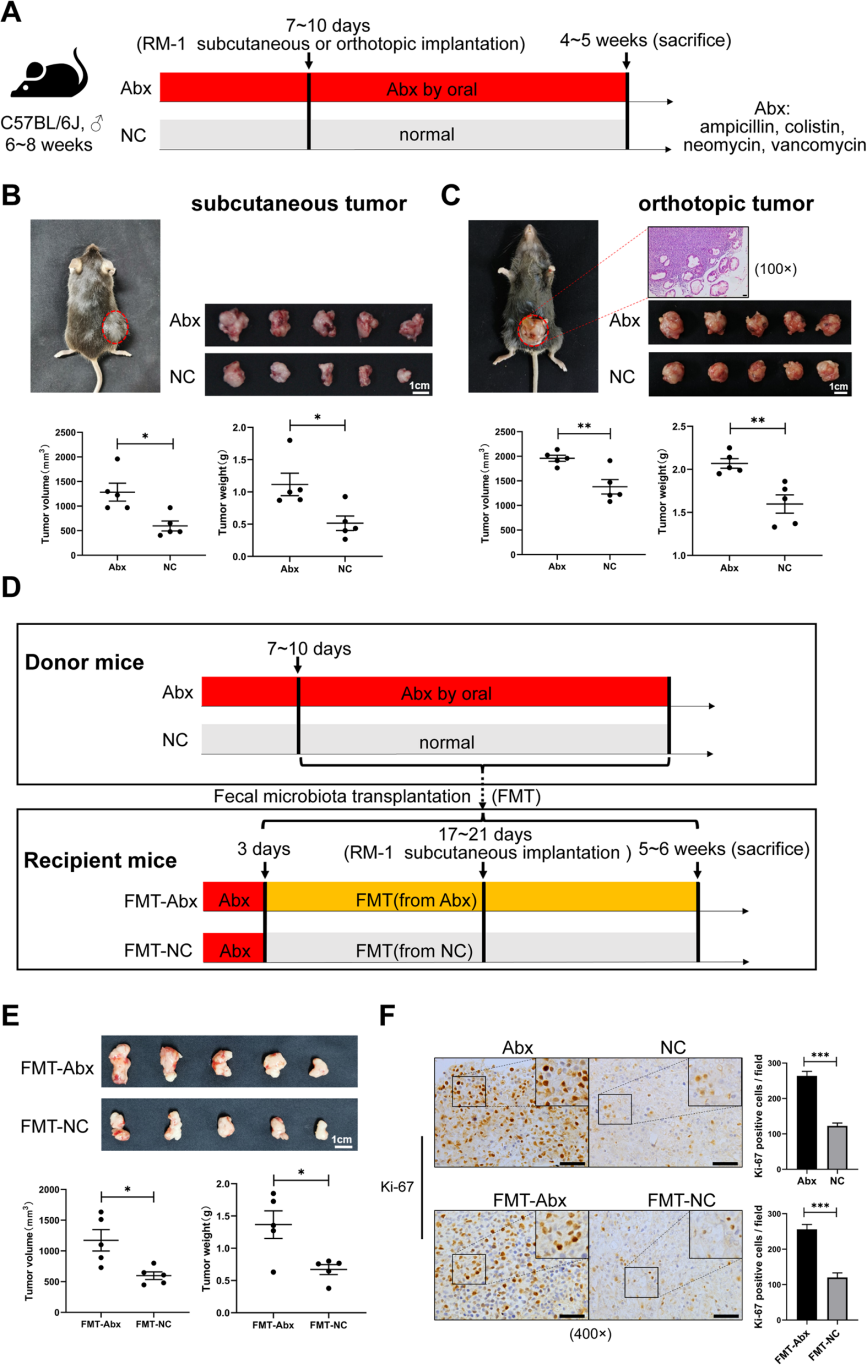
Antibiotic exposure promoted prostate cancer growth. **A** Flowchart of gut dysbiosis murine model establishment. **B** Images for mouse with subcutaneous tumor, tumors, and comparison of volume (mm^3^) and weight (g) for tumors in the Abx and NC group (*n* = 5). **C** Images for the mouse with orthotopic tumor (including section for HE staining, scale bar, 50 μm), tumors, and comparison of volume and weight for tumors in the Abx and NC group (*n* = 5). **D** Flowchart of FMT processing on mice. **E** Images for subcutaneous tumors and comparison of volume and weight for tumors in the FMT-Abx and FMT-NC group (*n* = 5). **F** Immunohistochemistry of tumor tissues for Ki-67-positive cell in the Abx and NC or the FMT-Abx and FMT-NC group (scale bar, 50 μm). Statistical significance was assessed by unpaired Student’s *T*-test. *p < 0.05, **p < 0.01, ***p < 0.001

A gut dysbiosis murine model was established by oral intake of antibiotics. C57BL/6J mice were continuously given water supplemented with broad-spectrum antibiotics (low intestinal absorption), including ampicillin, colistin, neomycin, and vancomycin (referred to henceforth as Abx), and prostate cancer tumor transplantation was performed with RM-1 (a murine CRPC cell line) subsequently (Fig. 1A). After a feeding period of 4–5 weeks, compared to the negative control (NC) group, antibiotic intervention effectively augmented subcutaneous tumor volume (*p* < 0.05) and weight (*p* < 0.05) (Fig. 1B). The same result was also observed with orthotopically transplanted tumors (Fig. 1C). In addition, in order to determine whether the gut microbiota was responsible for it, FMT was conducted (Fig. 1D). The results showed that, the subcutaneous tumor volume (*p* < 0.05) and weight (*p* < 0.05) were enhanced in the FMT-Abx group compared to the FMT-NC group (Fig. 1E). Consistently, immunohistochemical analysis of tumor tissues showed that the number of Ki-67-positive tumor cells was upregulated in the Abx and FMT-Abx groups (Fig. 1F). These results suggested that antibiotic exposure promoted prostate cancer growth in mice, which was associated with disturbing the gut microbiota.

### *Proteobacteria* was significantly enriched after antibiotic exposure

**Fig. 2:**
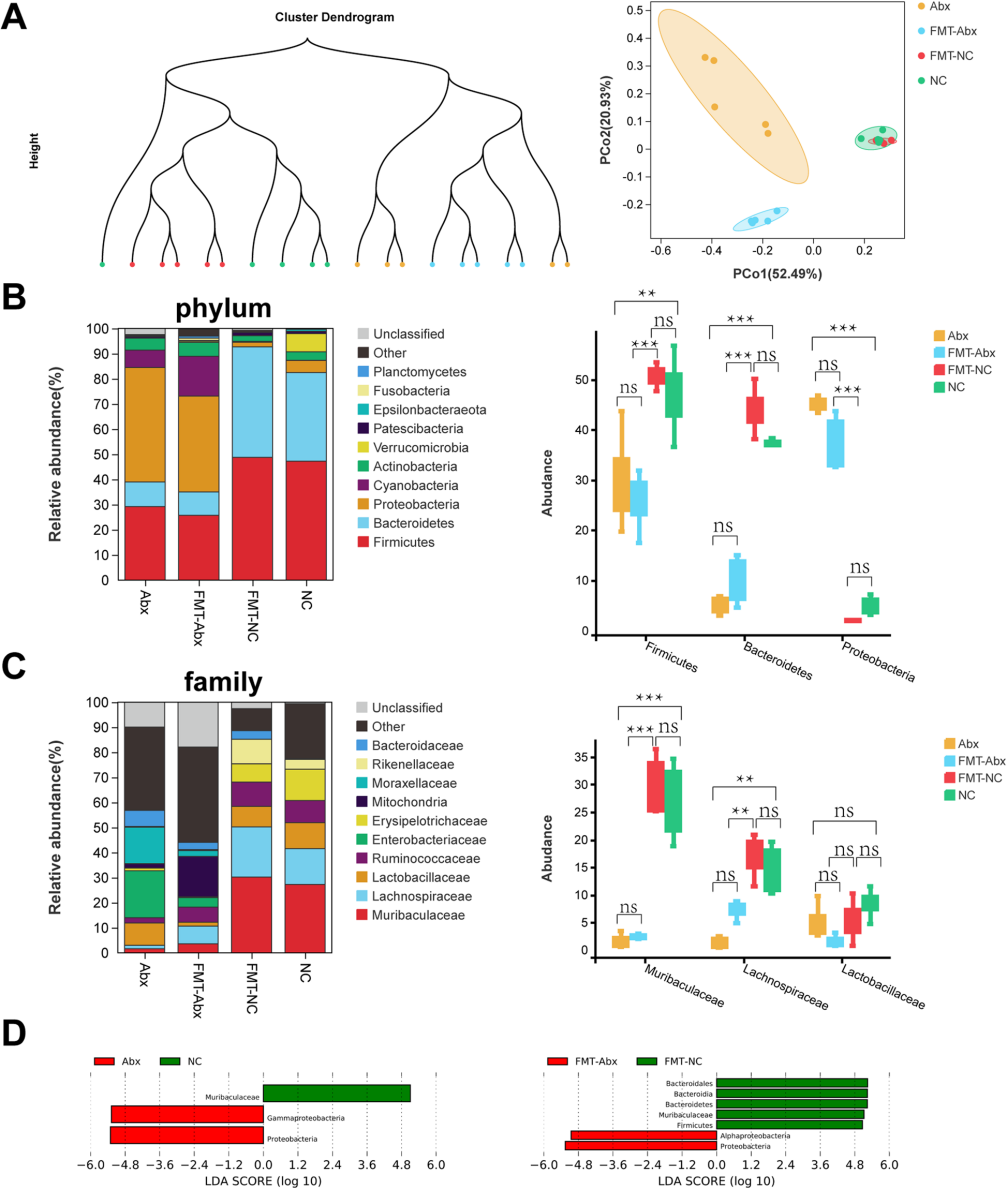
Composition of gut microbiota community in mice. **A** Cluster dendrogram and principal co-ordinates analysis (PCoA) of weighted_unifrac in four groups. **B** Composition of gut microbiota community (top 10) at the phylum level and the distribution of *Bacteroidetes*, *Firmicutes*, and *Proteobacteria* among four groups. **C** Composition of gut microbiota community (top 10) at the family level and the distribution of *Muribaculaceae*, *Lachnospiraceae*, and *Lactobacillaceae* among four groups. **D** LDA effect size (LefSe) demonstrating taxa with significantly different abundance (phylum to species, LDA score > 5). LDA (linear discriminant analysis). Statistical significance was assessed by Tukey-HSD in one-way ANOVA. *p < 0.05, **p < 0.01, ***p < 0.001

To investigate the composition of the gut microbiota, fecal samples from mice, including the Abx, FMT-Abx, NC, and FMT-NC groups, were collected and subjected to 16S rRNA sequencing. The Shannon and Simpson indices were applied to describe within-group species richness and evenness (α-diversity). The results showed that, compared to the Abx group, the Shannon index in the FMT-Abx was increased, but the Simpson index was not (Fig. S1A). Cluster dendrogram and principal co-ordinates analysis (PCoA), which are usually used to describe the variation in species abundance distribution between samples (β-diversity), revealed that there were similar bacterial community compositions between the Abx and FMT-Abx groups and between the NC and FMT-NC groups (Fig. 2A, Table S2).

Next, gut microbiota profiles of the four groups were assessed at various taxonomic levels. We found that the relative abundance of *Bacteroidetes* and *Firmicutes* were dominant in the NC and FMT-NC groups at the phylum level, while that of *Proteobacteria* was significantly enriched in the Abx and FMT-Abx groups (Fig. 2B). A remarkable increase in the relative abundance of the *Proteobacteria* phylum is considered a sign of an imbalanced gut microbiota because it only composes a minor proportion of natural gut microbes [29]. Meanwhile, we found that, at the family level, *Muribaculaceae* and *Lachnospiraceae* were significantly decreased in the Abx and FMT-Abx groups (Fig. 2C). To determine the biomarker species in each group, linear discriminant analysis effect size (LefSe) was calculated, and we found that *Proteobacteria* was the highest scoring species both in the Abx and FMT-Abx groups (LDA score > 5) (Fig. 2D). In summary, antibiotic exposure significantly altered the composition of the gut bacterial community. The relative abundance of *Proteobacteria* was significantly increased, which is considered to be a signature of gut dysbiosis.

### Intratumoral LPS activated NF-κB-IL6-STAT3 axis

**Fig. 3:**
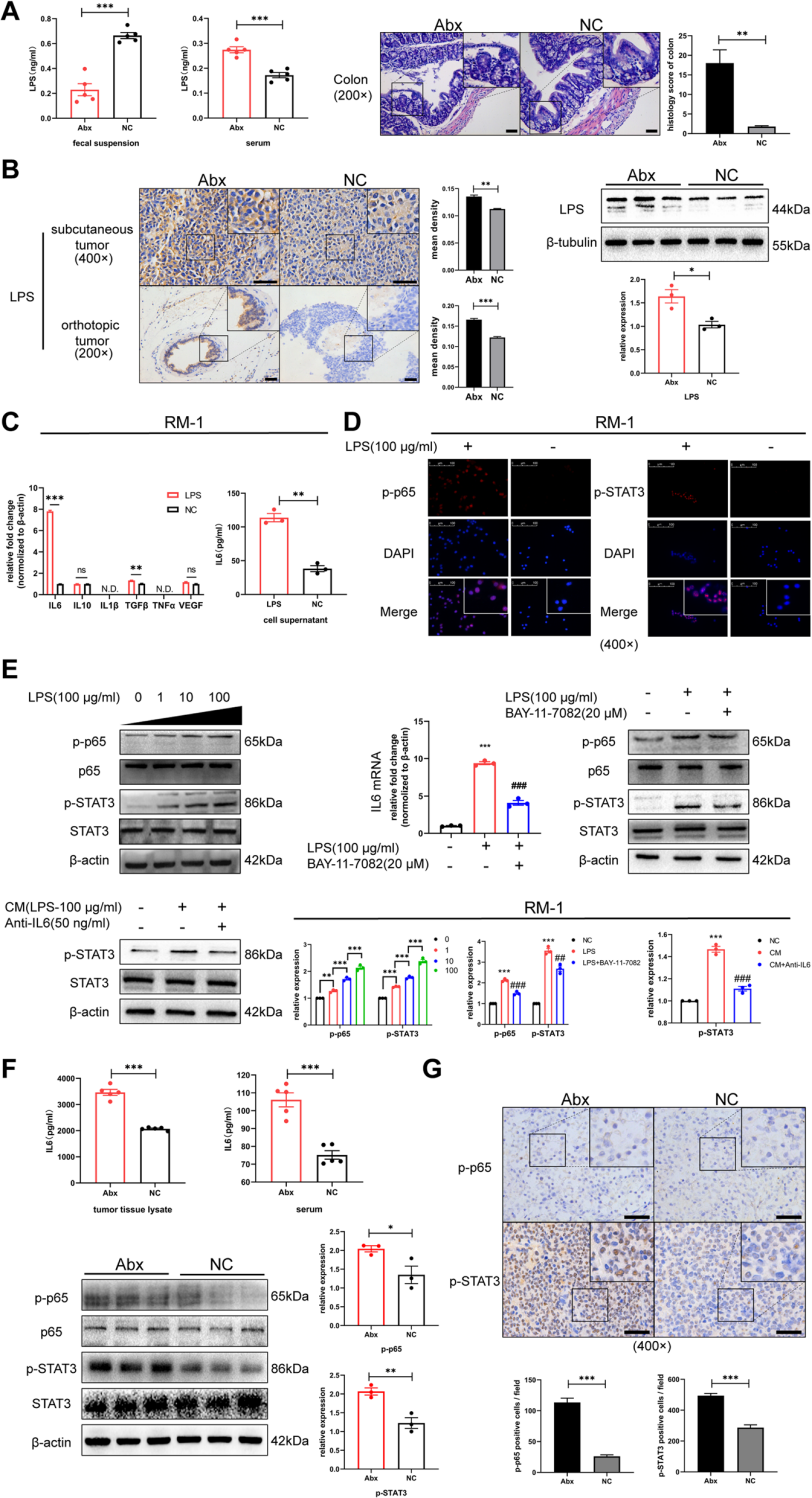
Intratumoral LPS activated NF-κB-IL6-STAT3 axis. **A** LPS levels in mouse feces and serum by ELISA; HE staining (scale bar, 50 μm) and histology score for colon tissue in the Abx and NC group. **B** Immunohistochemistry (scale bar, 50 μm) for LPS in subcutaneous and orthotopic tumor tissues and western blot of intratumoral LPS levels from three biological duplications for the Abx and NC group. **C** Transcription levels of cytokines by RT-qPCR and protein levels of IL6 in cell supernatant by ELISA in RM-1 cultured with LPS (100 μg/ml) for 24 h. **D**, **E** Immunofluorescence (scale bar, 100 μm) for p-p65 and p-STAT3 in RM-1 cultured with or without LPS for 24 h; western blot of relative proteins for RM-1 cultured with LPS at different concentrations for 24 h; transcription levels of IL6 by RT-qPCR and western blot of relative proteins in RM-1 cultured with LPS or LPS with BAY-11-7082 for 24 h; protein levels of p-STAT3 and STAT3 in RM-1 cultured with CM or CM with antibody-IL6 for 24 h. **F**, **G** IL6 levels in tumor tissue lysate and serum by ELISA. Western blot of relative proteins in tumor from three biological duplications. Immunohistochemistry of tumor tissues for p-p65- and p-STAT3-positive cell (scale bar, 50 μm). Statistical significance was assessed by unpaired Student’s *T*-test or LSD in one-way ANOVA. *p < 0.05, **p < 0.01, and ***p < 0.001: compared to the NC group; #*p* < 0.05, ##*p* < 0.01, and ###*p* < 0.001: compared to the LPS or CM group

In 2020, an analysis of the intratumoral bacterial community in pan-cancer showed that LPS, a product derived from Gram-negative bacteria as a critical inflammation-inducing factor, is detectable in a variety of human tumors, but its source still needs to be unraveled [30]. Based on the foregoing sequencing result, we hypothesized that the intestinal barrier was damaged under gut dysbiosis (characterized by the enrichment of *Proteobacteria*), and LPS was released into circulation, which then reached the tumor. To prove it, ELISA was conducted, and the results showed that although LPS levels in feces from the NC group were significantly higher compared to those from the Abx group, there was an opposite trend in serum. Additionally, a certain degree of damage was observed in the colon from the Abx group (Fig. 3A). These results indicated there was a leaky gut in mice from the Abx group. Moreover, LPS was significantly increased in tumors from the Abx group (Fig. 3B).

As mentioned above, LPS is known to trigger an inflammatory reaction by stimulating multiple cytokine secretion via activating the TLR4-NF-κB pathway. Recombinant LPS from *Escherichia coli* was used for cell culturing (RM-1), and transcription levels of cytokines were detected by RT-qPCR. We found that the relative fold change of IL6 was remarkably higher than those of other cytokines. Subsequently, ELISA confirmed that the protein levels of IL6 in the cell supernatant were also significantly increased (*p* < 0.01, Fig. 3C). Since IL6 is an activator of multiple signaling pathways, which can affect the expression level of key proteins of signaling pathways, and the Rever Phase Protein Array (RPPA) in LinkedOmics [21] provides relevant protein data of prostate cancer tissues, we select it to explore the role of IL6 in prostate cancer. As a result, we found a positive correlation between IL6 and p-STAT3 (Tyr705) through the RPPA and Gene Set Enrichment Analysis (GSEA) showed upregulation of the JAK-STAT pathway (Fig. S2A). Meanwhile, a positive correlation between the expression levels of p-STAT3 and p-p65 (the main transcription subunit of NF-κB) was found (Fig. S2B). Correspondingly, we showed that p-p65 and p-STAT3 were increased in the RM-1 nucleus with LPS (Fig. 3D) and were presented in a dose-dependent manner (Fig. 3E) in vitro. In the presence of an inhibitor for p65 phosphorylation (BAY-11-7082), the transcription levels of IL6 were diminished, as well as the protein levels of p-p65 and p-STAT3. To further confirm the role and effect manner of IL6, we created a conditioned medium (CM) by collecting the cell supernatant generated from tumor cells cultured with LPS. Compared to CM only, p-STAT3 was attenuated after adding the IL6 antibody to CM (Fig. 3E). To sum up, IL6, which was increased under activation of NF-κB by LPS, activated STAT3 in an autocrine manner. Consistent with this, IL6 concentrations in tumor tissue lysate and serum were increased in the Abx group compared to the NC group, as detected by ELISA. The same results were also observed with p-p65 and p-STAT3, as indicated by western blot and immunohistochemical analysis (Fig. 3F, G). In summary, intratumoral LPS was elevated under the upregulation of gut permeability, and it activated the NF-κB-IL6-STAT3 axis for prostate cancer in gut dysbiosis mice.

### Activation of the IL6-STAT3 pathway facilitated prostate cancer proliferation and docetaxel chemoresistance in gut dysbiosis mice

**Fig. 4:**
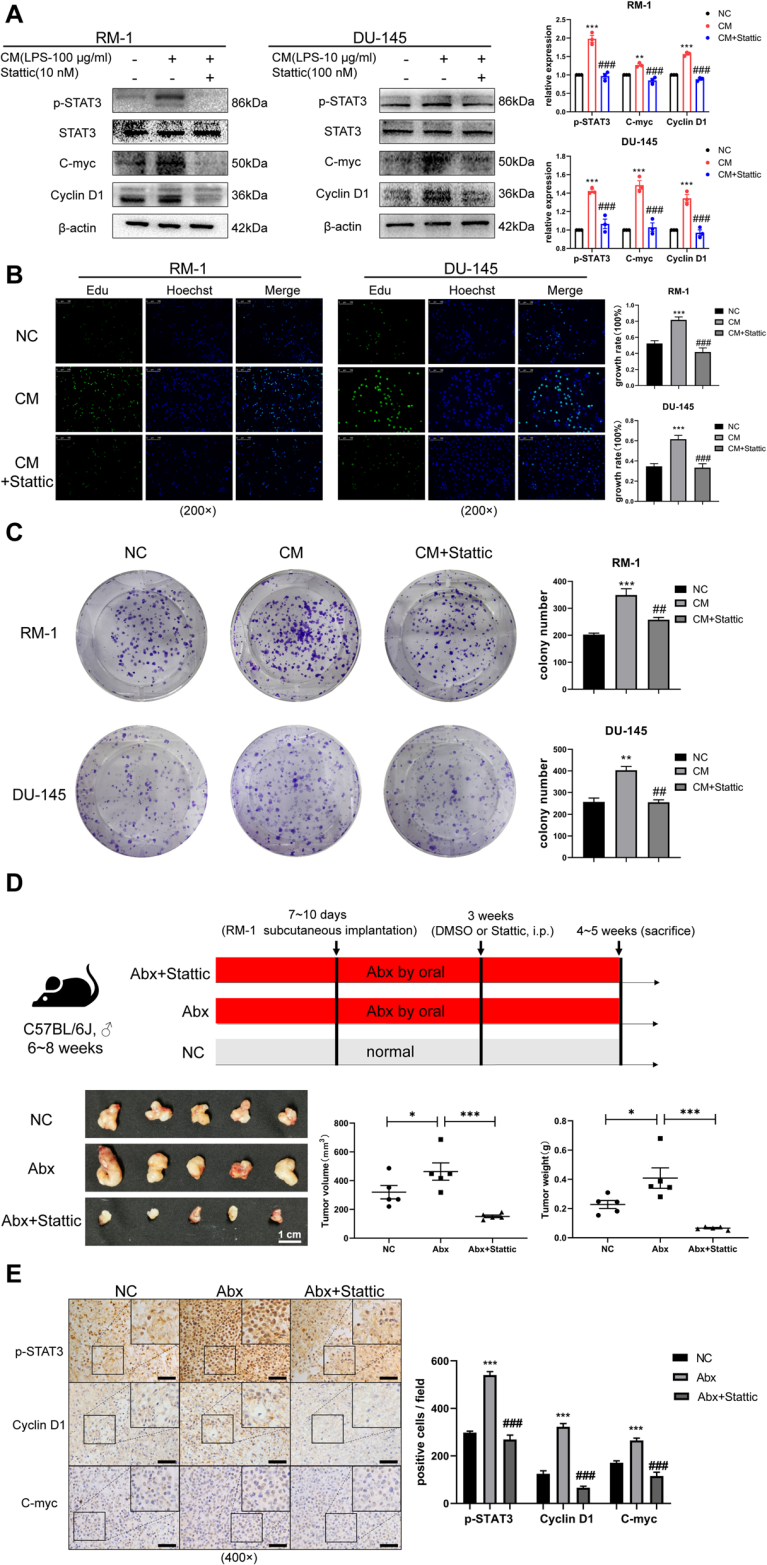
The IL6-STAT3 pathway promoted prostate cancer proliferation. **A** Western blot of relative proteins in RM-1 and DU-145 cultured with CM or CM with Stattic for 24 h. **B**, **C** Edu (scale bar, 100 μm) and clone formation assay were conducted on RM-1 and DU-145 under condition as described. **D** Flowchart of the NC, Abx, and Abx+Stattic groups for in vivo study. Relevant tumor images and comparison of volume and weight for tumors in three groups (*n* = 5). **E** Immunohistochemistry of tumor tissues for p-STAT3-, c-myc-, and cyclin D1-positive cell in three groups (scale bar, 50 μm). Statistical significance was assessed by LSD in one-way ANOVA. *p < 0.05, **p < 0.01, and ***p < 0.001: compared to the NC group; #*p* < 0.05, ##*p* < 0.01, and ###*p* < 0.001: compared to the CM or Abx group

The IL6-STAT3 pathway has been validated as capable of promoting progression in a variety of malignancies, and it is associated with tolerance for docetaxel, 5-fluorouracil, and other clinical chemotherapy drugs [31–33]. RM-1 and DU-145 (a human CRPC cell line) were chosen for in vitro experiments subsequently. Consistent with the findings above, western blot showed that p-STAT3 was increased in cells cultured with CM but diminished in the presence of the STAT3 phosphorylation inhibitor (Stattic) [34]. STAT3 downstream proliferation-related genes, such as cyclin D1 and c-myc, were altered accordingly both in RM-1 and DU-145 (Fig. 4A). Meanwhile, Edu (5-ethynyl-2′-deoxyuridine) detection assay and clone formation assay were used to detect the prostate cancer cell proliferation ability in vitro and the result indicated that inhibition of the IL6-STAT3 pathway suppressed prostate cancer cell line proliferation (Fig. 4B, C). During an in vivo experiment, compared to the Abx group, the tumor volume (*p* < 0.001) and weight (*p* < 0.001) in the Abx+Stattic group were significantly reduced (Fig. 4D). Immunohistochemical analysis of tumor tissues demonstrated that, compared to the NC group, p-STAT3, cyclin D1, and c-myc were enhanced in the Abx group but were decreased in the Abx+Stattic group (Fig. 4E).

**Fig. 5:**
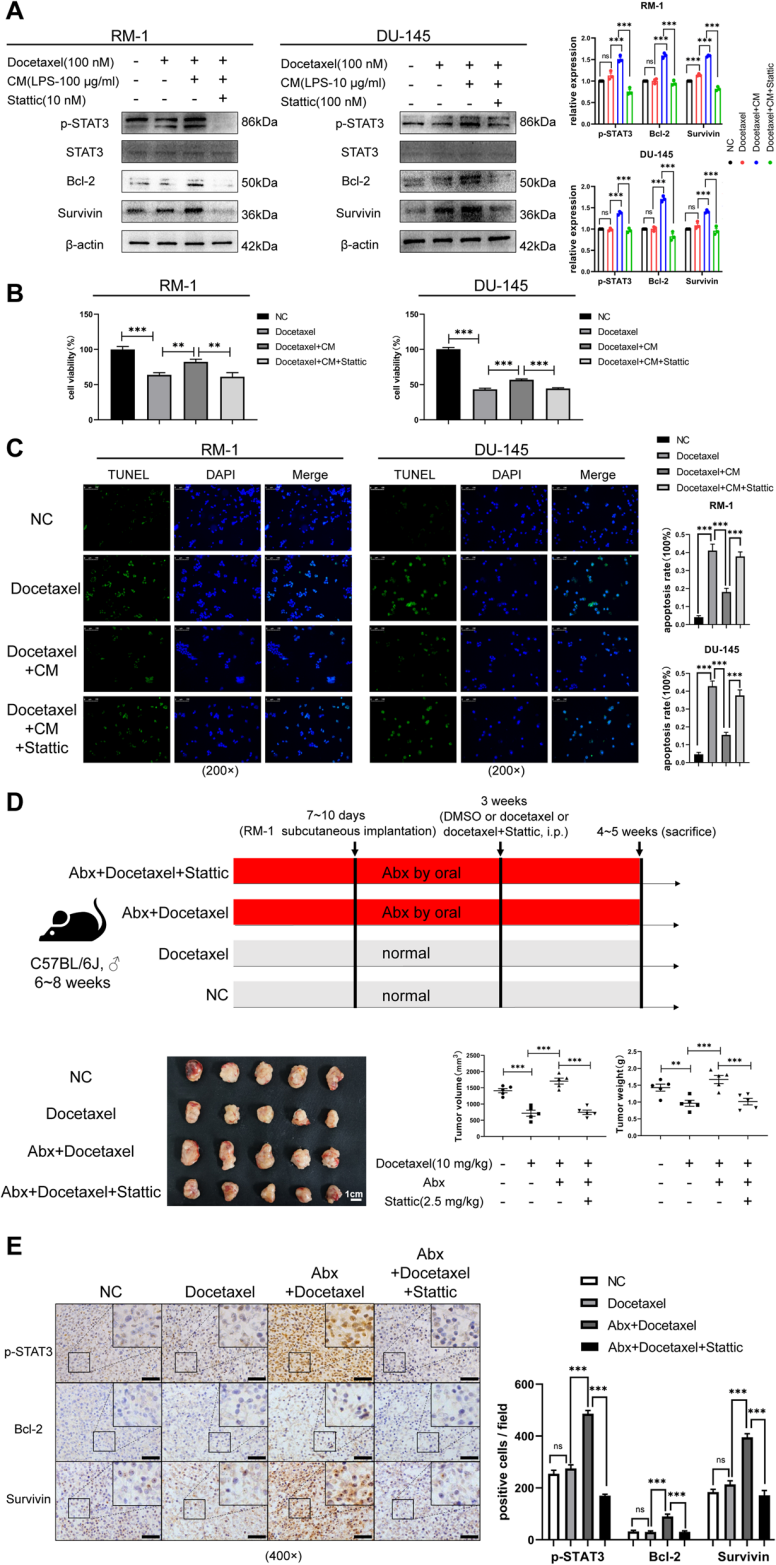
The IL6-STAT3 pathway promoted prostate cancer docetaxel chemoresistance. **A** Western blot of relative proteins in RM-1 and DU-145 cultured with different conditions. **B**, **C** Cell viability and TUNEL assay (scale bar, 100 μm) were conducted on RM-1 and DU-145 under condition as described. **D** Flowchart of the NC, Docetaxel, Abx+Docetaxel, and Abx+Docetaxel+Stattic groups for in vivo study. Relevant tumor images and comparison of volume and weight for tumors in four groups (*n* = 5). **E** Immunohistochemistry of tumor tissues for p-STAT3-, Bcl-2-, and survivin-positive cell in four groups (scale bar, 50 μm). Statistical significance was assessed by LSD in one-way ANOVA. *p < 0.05, **p < 0.01, ***p < 0.001

Docetaxel, as a first-line treatment for CRPC, is inevitably leading to drug resistance in the clinic, and one of the underlying mechanisms of this may be activation of the IL6-STAT3 pathway [35]. Two cell lines, RM-1 and DU-145, were cultured for 24 h under three conditions: blank, CM alone, and CM with Stattic. Following the above treatments, we added docetaxel to the media for 24-h cultivation. Similarly, western blot showed that p-STAT3 was increased in cells from the CM-culturing system but was decreased with Stattic, as were anti-apoptotic proteins, such as Bcl-2 and survivin. Interestingly, treatment with docetaxel alone was likely to increase the levels of these (Fig. 5A). Cell viability and TUNEL assay suggested that suppressing the IL6-STAT3 pathway ameliorated tolerance for docetaxel in prostate cancer cell line (Fig. 5B, C). Consistently, in vivo experimentation further confirmed this conclusion. Docetaxel significantly reduced tumor volume (*p* < 0.001) and weight (*p* < 0.01) in mice fed normally but failed to do so in gut dysbiosis ones. However, combining docetaxel and Stattic significantly reduced both tumor volume (*p* < 0.001) and weight (*p* < 0.001) (Fig. 5D). Immunohistochemical analysis of tumor tissues showed that p-STAT3, Bcl-2, and survivin were changed accordingly (Fig. 5E). These results indicated that the IL6-STAT3 pathway was activated in tumors from gut dysbiosis mice and facilitated proliferation and docetaxel chemoresistance for prostate cancer.

### Clinical characteristics and gut microbiota profile in patients

16S rRNA sequencing data of the gut microbiota in prostate cancer patients have been reported, but few species that can be used as biomarkers were found [36, 37]. The results above suggested that gut dysbiosis contributes to prostate cancer progression in mice. However, understanding the effects of gut microbiota on the risk of prostate cancer progression requires further evidence from clinical patient samples. Patients with benign prostatic hyperplasia (BPH, *n* = 20), non-metastatic prostate cancer (*n* = 10), and metastatic prostate cancer (*n* = 5) were recruited to this study. Patient characteristics and TNM stage are shown in Tables 1 and 2. There was no significant difference in various variables, aside from prostate-specific antigen (PSA) level (*p* < 0.001). The Gleason score group of patients with metastatic prostate cancer was higher than those of patients with non-metastatic prostate cancer, and prognostic grouping (as assessed by the AJCC group) of patients with metastatic prostate cancer was worse.

**Table 1:** Baseline characteristics of patients with benign prostatic hyperplasia, non-metastatic prostate cancer or metastatic prostate cancer

Variables	BPH (*n* = 20)	nmPCa (*n* = 10) *n* (%) or median (IQR)	mPCa (*n*=5)	*P* value
Age (years)	70.5 (63–76.75)	69.5 (66.5–75)	68 (65–74)	0.21
BMI (kg/m^2^)	22.11 (19.06–26.58)	25.21 (23.26–27.87)	23.88 (19.80–26.67)	0.387
PSA (ng/mL)	4.12 (2.14–10.30)	17.36 (12.98–79.74)	67.66 (42.55–4146.35)	<0.001^a^
IL6^b^ (pg/mL)	8 (3.58–10.65)	15.5 (10.65–21.65)	13.5 (-)	0.087
LUTS	18 (90)	7 (70)	3 (60)	0.21
Gleason score group				
G1 (3+3)		1 (10)		
G2 (3+4)		1 (10)		
G3 (4+3)		2 (20)		
G4 (4+4, 3+5, 5+3)		4 (40)		
G5 (4+5, 5+4, 5+5)		2 (20)	5 (100)	

*BPH* benign prostatic hyperplasia, *nmPCa* non-metastatic prostate cancer, *mPCa* metastatic prostate cancer, *IQR* interquartile range, *PSA* prostate-specific antigen, *LUTS* lower urinary tract symptoms

^a^Kruskal-Wallis test: BPH vs nmPCa (*p* = 0.005), BPH vs mPCa (*p* < 0.001), mPCa vs nmPCa (*p* = 0.657)

^b^Available data: BPH (*n* = 12), nmPCa (*n* = 6), mPCa (*n* = 2). Analysis was performed by the total available patients

**Table 2:** TNM stage of patients with non-metastatic prostate cancer or metastatic prostate cancer

Variables	nmPCa (*n* = 10)	mPCa (*n* = 5)
	*n* (%)	
T^a^		
Tx		1 (20)
T1		
T2	5 (50)	
T3	5 (50)	2 (40)
T4		2 (40)
N		
Nx		1 (20)
N0	9 (90)	1 (20)
N1	1 (10)	3 (60)
M		
M0	10 (100)	
M1		5 (100)
AJCC group (I–IV)^b^		
I		
IIA		
IIB		
IIC	3 (30)	
IIIA		
IIIB	4 (40)	
IIIC	2 (20)	
IVA	1 (10)	
IVB		5 (100)

*nmPCa* non-metastatic prostate cancer, *mPCa* metastatic prostate cancer

^a^Including clinical and pathological T

^b^2017 AJCC (American Joint Committee on Cancer) Eighth Edition

**Fig. 6:**
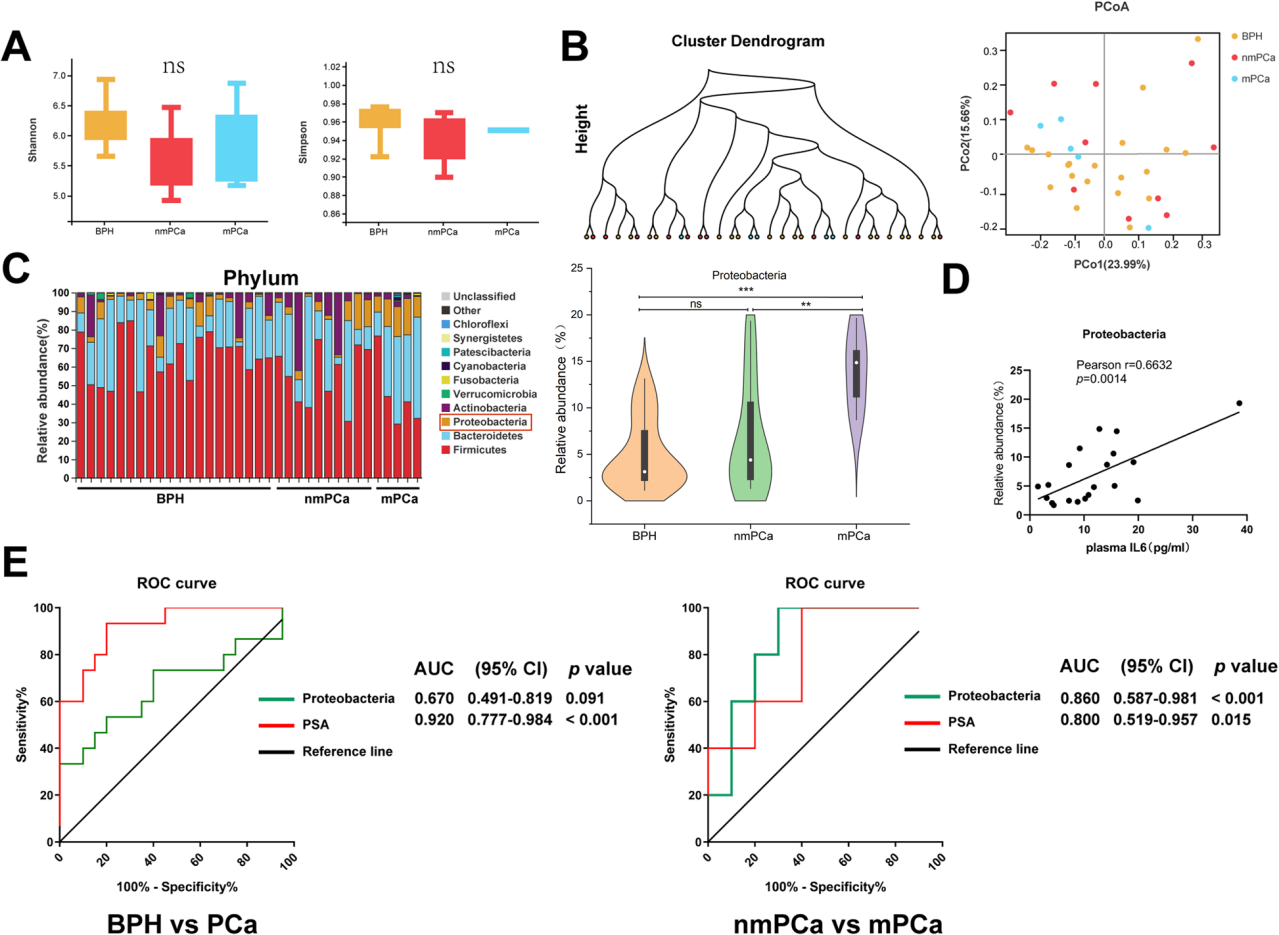
Clinical characteristics and gut microbiota profile in patients. **A**, **B** Shannon and Simpson indices, and cluster dendrogram and principal co-ordinates analysis (PCoA) of weighted_unifrac in three groups. **C** Composition of gut microbiota community (top 10) at the phylum level, and the distribution of *Proteobacteria* in three groups. **D** Correlation analysis between plasma IL6 level and the relative abundance of *Proteobacteria* in patients (*n* = 20). **E** ROC curves for prostate cancer vs BPH or non-metastatic prostate cancer vs metastatic prostate cancer. PSA level was used as a standard predictive marker. ROC values were conducted by MedCalc version 20. Statistical significance was assessed by Tukey-HSD and LSD in one-way ANOVA. *p < 0.05, **p < 0.01, ***p < 0.001

Fecal samples from patients were subjected to 16S rRNA sequencing, and we found that α- and β-diversity showed no significant difference among the three groups (Fig. 6A, B, Table S2). Of note, bacterial composition at the phylum level indicated that the relative abundance of *Proteobacteria* in patients with metastatic prostate cancer was higher than that in those with non-metastatic prostate cancer or BPH, and a difference was also observed between prostate cancer and BPH patients (Fig. 6C, S3A). *Gammaproteobacteria* (class), *Enterobacteriales* (order), *Enterobacteriaceae* (family), and *Escherichia* (genus), which are typical species belonging to the *Proteobacteria* phylum, were also more common in patients with metastatic prostate cancer, but without a statistically significant difference, except regarding *Gammaproteobacteria* (Fig. S3B). Unfortunately, there were no biomarker species identified by LefSe. Next, correlation analysis was conducted between *Proteobacteria* and multiple clinical parameters, and the results showed that the relative abundance of *Proteobacteria* was positively correlated with plasma IL6 level, regional lymph node metastasis status, and distant metastasis status (Fig. 6D, Table 3). Finally, receiver operating characteristic (ROC) curves revealed that, although *Proteobacteria* failed to distinguish prostate cancer from BPH, it had better potential in predicting distant metastasis than PSA level (tended to be higher in patients with metastatic prostate cancer) (area under the ROC curve, 0.860; *p* < 0.001) (Fig. 6E).

**Table 3:** Spearman correlation between the relative abundance of *Proteobacteria* and various parameters in prostate cancer patients

Variables	T (*n* = 14)	N (*n* = 14)	M (*n* = 15)	GS group (*n* = 15)	AJCC group (*n* = 15)
rho	0.166	0.667	0.589	0.347	0.467
*p* value	0.570	**0.009**	**0.021**	0.206	0.080

*GS group* Gleason score group

## Discussion

Prostate cancer is a multifactorial and complex disease involving factors from the genome, environment, and immunity [38]. As an external environmental factor, gut microbiota is the largest source of human microorganisms, which has been proved to contribute to the progression of various malignancies. However, the influence of the gut microbiota on prostate cancer is not well understood. In this research, we found that gut dysbiosis caused by antibiotic exposure resulted in enrichment of intestinal *Proteobacteria* and elevation of gut permeability and intratumoral LPS, promoting prostate cancer proliferation and docetaxel chemoresistance via NF-κB-IL6-STAT3 axis in C57BL/6J mice. Meanwhile, 16S rRNA sequencing of clinical fecal samples showed that the relative abundance of *Proteobacteria* was elevated in patients with metastatic prostate cancer and was positively correlated with multiple clinical parameters.

The role of antibiotic exposure in tumor progression is debatable. An initial study reported that oral broad-spectrum antibiotics restrained the growth of subcutaneous transplanted tumors, such as colon cancer, pancreatic tumor, and melanoma, and their liver metastasis capacity was also inhibited [39]. Conversely, broad-spectrum antibiotics accelerated the process of colitis-associated cancer development in mice [40]. In our study, gut dysbiosis caused by antibiotic exposure promoted prostate cancer growth, regardless of whether the tumor was subcutaneous or orthotopic, and FMT proved that it was mediated by the gut microbiota. This result could be attributed to the type of tumor, the murine model used in the study, the type of antibiotics used, and the specific damage done to different species members of the gut microbiome under the specific antibiotics. We provided evidence that antibiotic exposure promoted tumor progression in this study, consistent with findings of other recent investigations [13–15].

Although both Shannon and Simpson indices are commonly used to describe species α-diversity, the Simpson index is more sensitive to dominant species and heavily weighted towards the most abundant species in the sample while being less sensitive to species richness. The Shannon index is more sensitive to species richness and weighted towards uncommon species [41]. There are similar dominant species (*Proteobacteria*) in the Abx and FMT-Abx group, so there is no difference in the Simpson index. On the other hand, α-diversity was decreased in the Abx group indicated by the Shannon index, but we cannot confirm whether the FMT-Abx group fully accepted the gut microbiota profile from the Abx group for 100% in the FMT process, especially for the uncommon species. We cannot rule out that the changes of the Shannon index came from this.

The steady state of gut microbiota is usually dominated by obligate anaerobic *Firmicutes* and *Bacteroidetes*, while the rising abundance of *Proteobacteria* (facultative anaerobic) is considered to be a sign of dysbiosis. Numerous studies have reported that it is a biomarker phylum of biomarker feature for IBD, obesity, diabetes, and other metabolic diseases [29]. 16S rRNA sequencing in our study showed that the relative abundance of *Proteobacteria* was significantly elevated after antibiotic exposure. This could be partly explained by the finding that antibiotic exposure to eliminate butyric-producing bacteria destroyed the butyrate-PPARγ signal axis in intestinal epithelial cells, enhancing colonic nitric acid and oxygen levels and resulting in maladjusted expansion of *Enterobacteriaceae* (*Proteobacteria* phylum) [42].

Organ-gut microbiota interactions involve direct or indirect contact. This study focused on the influence of the product (LPS), derived from gut microbes, on extra-intestinal tumors in an indirect way. The levels of fecal LPS suggested that the absolute number of gut bacteria in the Abx group was significantly lower than that in the NC group under antibiotic exposure, but colonic morphology and LPS levels in serum and tumor tissues implied that gut permeability was increased in gut dysbiosis mice. The decrease in SCFA-producing bacteria (such as *Lachnospiraceae*) caused by antibiotic exposure may be one of the reasons for the destruction of the intestinal barrier and the elevation of gut permeability. As energy substrates for colonic epithelial cells, SCFAs regulate cell proliferation, differentiation, and apoptosis, as well as the secretion of mucus and antimicrobial peptides, to maintain the integrity of the intestinal barrier [43].

Prostate cancer is an androgen-dependent malignancy in the initial stage, and androgen deprivation therapy (ADT) has been developed accordingly. However, prostate cancer demonstrates androgen-independent growth in the castration-resistant stage, involving various carcinogenic mechanisms. LPS, as a pathogen-associated molecular pattern (PAMP), stimulates the secretion of a series of inflammatory cytokines (mainly IL6 in this study) via the TLR4-NF-κB pathway. We found that intratumoral LPS activated the NF-κB-IL6-STAT3 axis with upregulation of downstream carcinogenic genes, contributing to the progression of prostate cancer. LPS also promotes tumor progression via enhancement of the epithelial-mesenchymal transformation (EMT) and angiogenesis [44].

Commensal bacteria affect chemotherapy effectiveness. Tumor-bearing mice treated with antibiotics for deleting intestinal bacteria or germ-free mice were resistant to cyclophosphamide, possibly owing to a weakened anti-tumor immune response [45]. Gemcitabine, a chemotherapeutic drug commonly used to treat pancreatic ductal adenocarcinoma (PDAC), was metabolized into its inactive form by *Gammaproteobacteria* residing in the tumor [46]. IL6-STAT3 has been reported to play a vital role in resistance to chemotherapy for multiple tumors, including prostate cancer. Nevertheless, our study is seemingly the first to report that gut dysbiosis activated the intratumoral NF-κB-IL6-STAT3 axis and was associated with docetaxel chemoresistance in prostate cancer. In vitro and in vivo experiments demonstrated that combining docetaxel with Stattic could ameliorate chemotherapy efficacy. This result is expected to provide a theoretical basis for overcoming clinical docetaxel resistance.

Furthermore, specific tumor-associated bacteria contribute to both diagnosis and prediction of disease progression. A previous study showed that unique microbial markers could be found in blood and tumor tissues, and microbial nucleic acids in plasma could be used to distinguish tumor patients from normal individuals [47]. Meanwhile, identifying biomarker species at different stages of tumor development may help to predict disease progression. *Fusobacterium nucleatum* was increased continuously from intramucosal carcinoma to more progressive stages in human specimens, and in vivo studies suggested that it played a vital role in the metastatic progression of colorectal and breast cancer [48–50]. We also found that the relative abundance of *Proteobacteria* in patients with metastatic prostate cancer was higher, which was positively correlated with multiple clinical parameters. *Proteobacteria* failed to distinguish prostate cancer from BPH in ROC curve analysis but sufficed to discern between metastatic and non-metastatic prostate cancer. It is therefore expected to be used as an auxiliary diagnostic or predictive approach for progressive prostate cancer. However, due to our small sample size and single-center analysis, it is necessary to verify our findings using larger sample sizes and multicenter studies.

## Conclusions

**Fig. 7:**
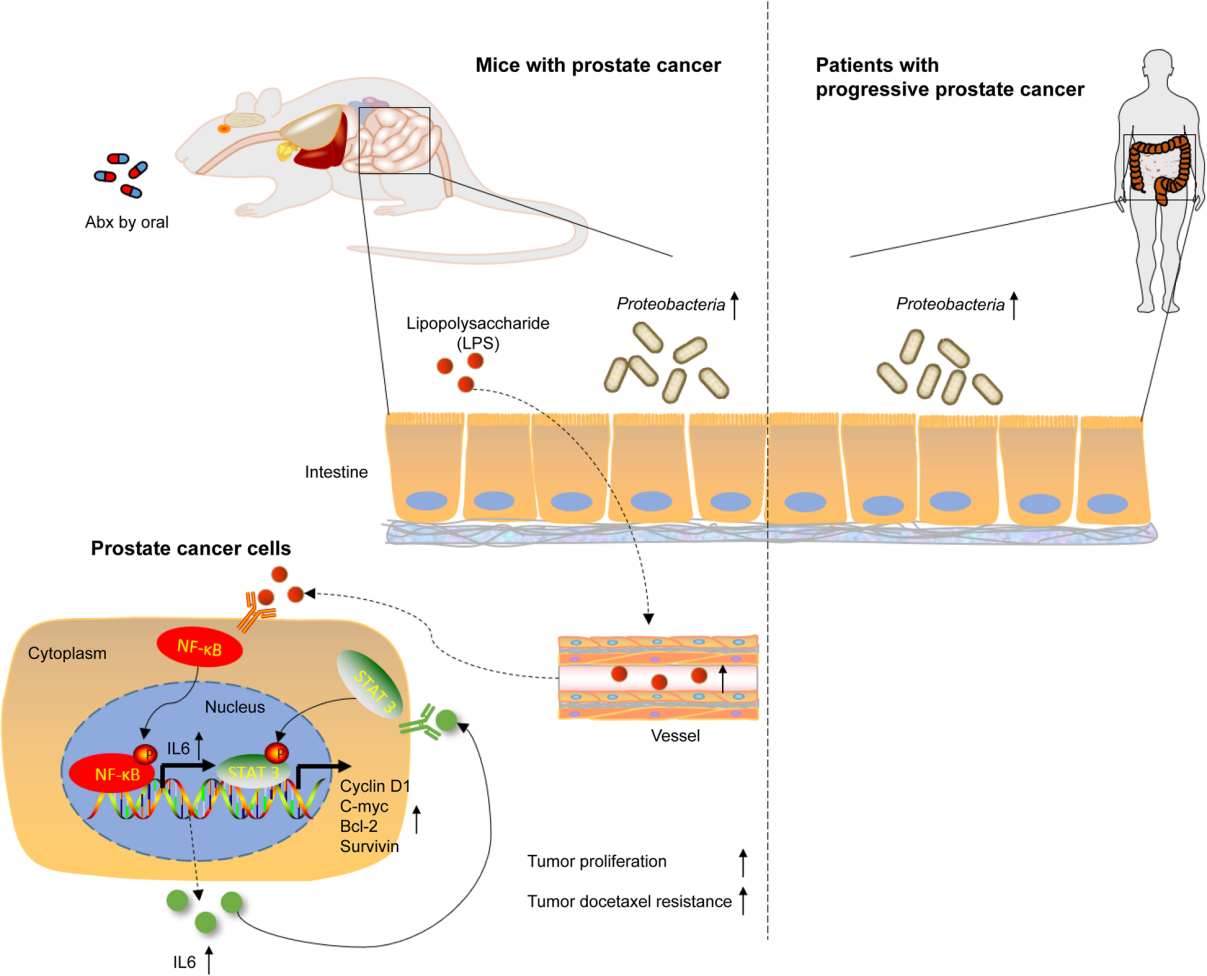
Schematic diagram. The schematic diagram is divided into three parts: (1) Continuously oral intake of Abx caused the enrichment of intestinal *Proteobacteria* in mice and intratumoral LPS was increased under gut leaky. (2) Intratumoral LPS activated the NF-κB-IL6-STAT3 axis, promoting prostate cancer proliferation and docetaxel chemoresistance. (3) *Proteobacteria* was enriched in patients with progressive prostate cancer. This diagram was performed by Science Slides 2016

In summary, we offer a new observation regarding the influence of gut microbiota on prostate cancer. Gut dysbiosis, characterized by the enrichment of *Proteobacteria* due to antibiotic exposure, resulted in the elevation of gut permeability and intratumoral LPS, which promoted the development of prostate cancer via the NF-κB-IL6-STAT3 axis in mice. Moreover, *Proteobacteria* may be used as an intestinal biomarker of progressive prostate cancer based on findings from patients (Fig. 7).

## Supplementary Information

**Additional file 1: Supplement Figure 1.** α-diversity for gut microbiota in mice. (A) Shannon and Simpson indices among four groups. Statistical significance was assessed by Tukey-HSD in one-way ANOVA. **p<0.01: compared to Abx group.

**Additional file 2: Supplement Figure 2.** The relationship among IL6, p-STAT3, and p-p65 in prostate cancer from LinkedOmics database for human. (A) IL6 associated genes from RPPA data for prostate cancer in LinkedOmics was shown by volcano plot (STAT3_pY705 framed by red box) and GSEA showed upregulation of JAK-STAT pathway. (B) Positive correlation between p-p65 and p-STAT3 expression for prostate cancer patient cohort (*n* = 341) in LinkedOmics.

**Additional file 3: Supplement Figure 3.** Composition of gut bacterial community in patients. (A) The distribution of *Proteobacteria* between benign prostatic hyperplasia and prostate cancer. (B) The distribution of *Gammaproteobacteria*, *Enterobacteriales*, *Enterobacteriaceae* and *Escherichia* among three groups. Statistical significance was assessed by unpaired Student’s T-test or LSD in one-way ANOVA. *p<0.05, **p<0.01, ***p<0.001.

**Additional file 4: Supplement Table 1.** The primer sequences used in the present research.

**Additional file 5: Supplement Table 2.** β-diversity for mice (above) and human (below) of 16S rRNA sequence.

**Additional file 6.** Human (phylum to species).

**Additional file 7.** Mouse (phylum to species).

**Additional file 8.** Sequencing data for fecal samples of both PCa patients and mice.

## Data Availability

The partial results generated during the current study are available in the LinkedOmics repository (available website: http://www.linkedomics.org/login.php). The 16S rRNA sequencing data used during the current study are available from the corresponding author on reasonable request.

## Abbreviations

ADT: Androgen deprivation therapy
AJCC: American Joint Committee on Cancer
AUC: Area under the curve
BPH: Benign prostatic hyperplasia
BMI: Body mass index
CI: Confidence interval
CM: Conditioned medium
CRPC: Castration-resistant prostate cancer
Edu: 5-Ethynyl-2′-deoxyuridine
ELISA: Enzyme-linked immunosorbent assay
EMT: Epithelial-mesenchymal transformation
FMT: Fecal microbiota transplantation
GSEA: Gene Set Enrichment Analysis
HE: Hematoxylin-eosin
IBD: Inflammatory bowel disease
ICI: Immune checkpoint inhibitor
LDA: Linear discriminant analysis
LefSe: Linear discriminant analysis effect size
LPS: Lipopolysaccharide
NC: Negative control
OD: Optical density
PAMP: Pathogen-associated molecular pattern
PCa: Prostate cancer
PCoA: Principal co-ordinates analysis
PDAC: Pancreatic ductal adenocarcinoma
PPARγ: Peroxisome proliferator-activated receptor γ
PRAD: Prostate adenocarcinoma
PSA: Prostate-specific antigen
ROC: Receiver operating characteristic
RPPA: Rever phase protein array
RT-qPCR: Real-time quantitative PCR
SCFAs: Short-chain fatty acids
SPF: Specific pathogen free
TUNEL: TdT-mediated dUTP Nick-End Labeling
