# Supplementary figures and images for: Gut dysbiosis promotes prostate cancer progression and docetaxel resistance via activating NF-κB-IL6-STAT3 axis
Source: Microbiome. 2022 Jun 16;10:94. doi: 10.1186/s40168-022-01289-w (PMC9202177; doi:10.1186/s40168-022-01289-w)

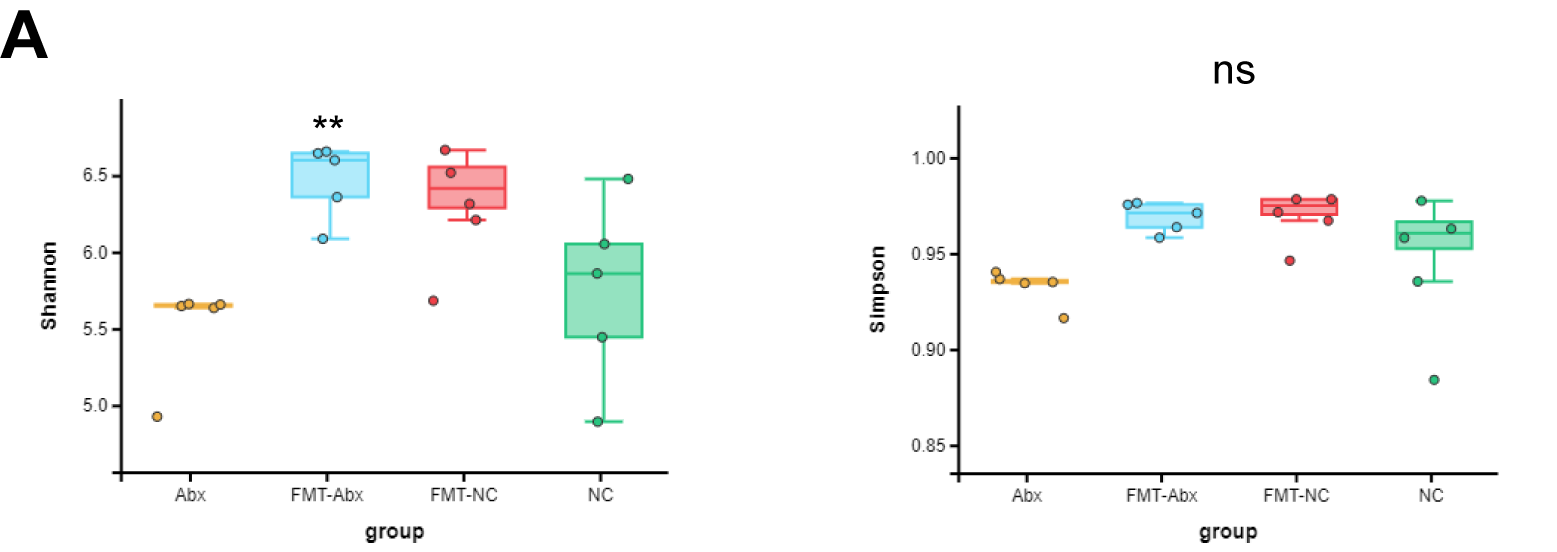

Supplement: Supplementary file 2 — Additional file 1: Supplement Figure 1. α-diversity for gut microbiota in mice. (A) Shannon and Simpson indices among four groups. Statistical significance was assessed by Tukey-HSD in one-way ANOVA. **p<0.01: compared to Abx group. [file 40168_2022_1289_MOESM1_ESM.tif]

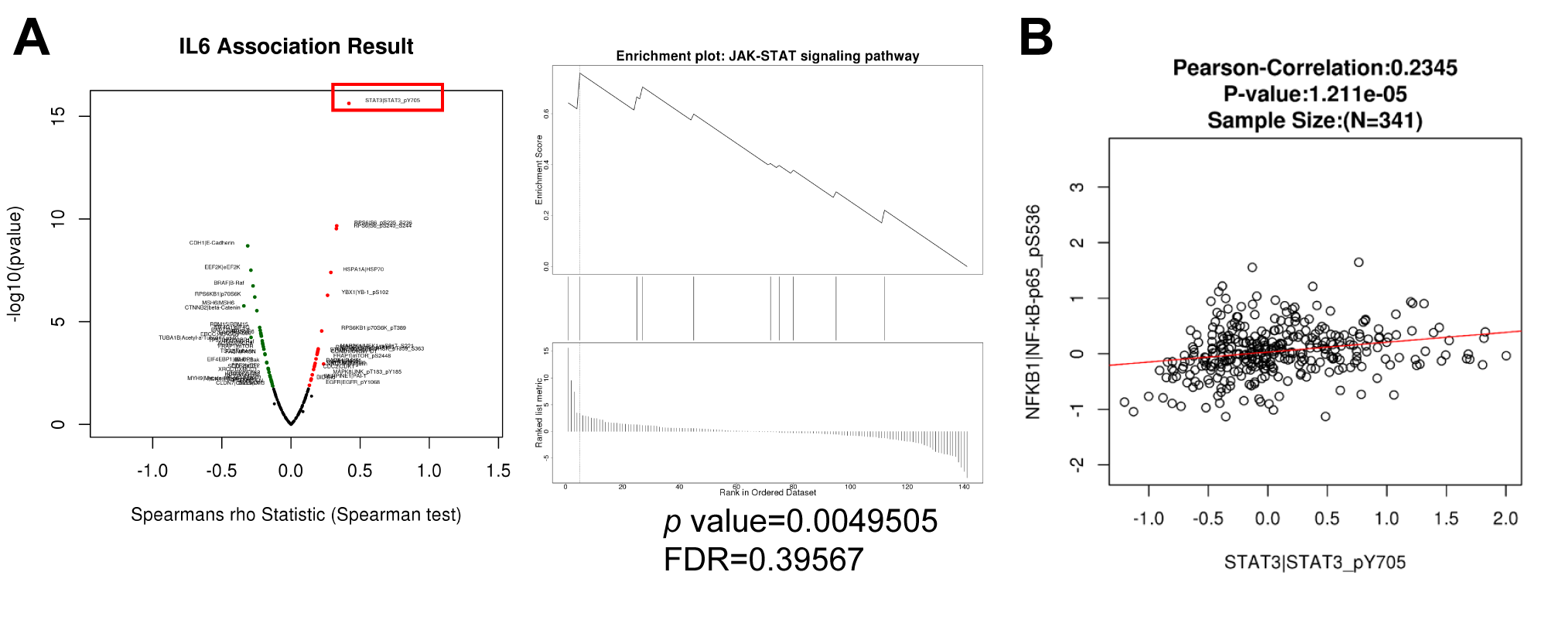

Supplement: Supplementary file 3 — Additional file 2: Supplement Figure 2. The relationship among IL6, p-STAT3, and p-p65 in prostate cancer from LinkedOmics database for human. (A) IL6 associated genes from RPPA data for prostate cancer in LinkedOmics was shown by volcano plot (STAT3_pY705 framed by red box) and GSEA showed upregulation of JAK-STAT pathway. (B) Positive correlation between p-p65 and p-STAT3 expression for prostate cancer patient cohort (n = 341) in LinkedOmics. [file 40168_2022_1289_MOESM2_ESM.tif]

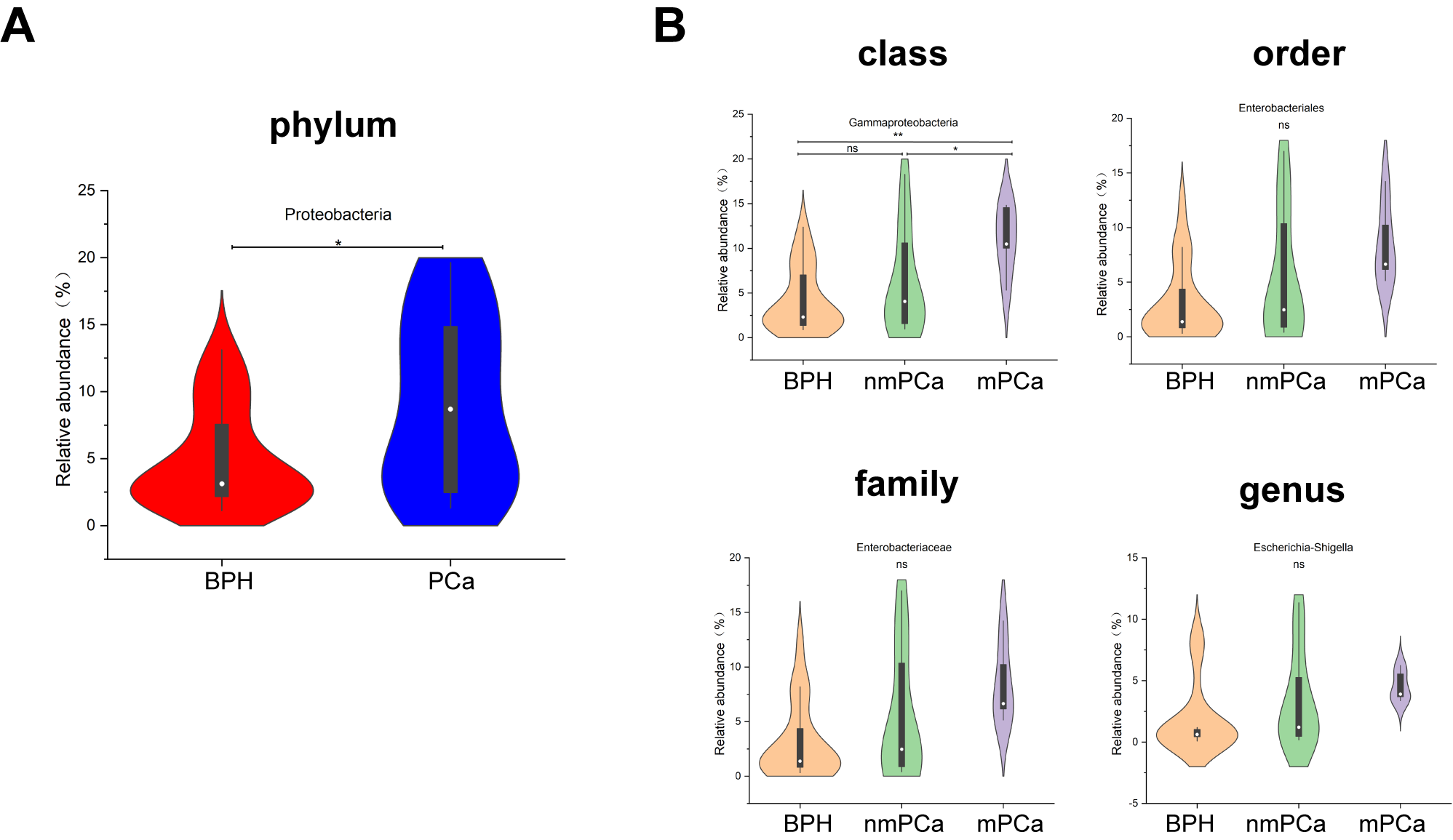

Supplement: Supplementary file 4 — Additional file 3: Supplement Figure 3. Composition of gut bacterial community in patients. (A) The distribution of Proteobacteria between benign prostatic hyperplasia and prostate cancer. (B) The distribution of Gammaproteobacteria, Enterobacteriales, Enterobacteriaceae and Escherichia among three groups. Statistical significance was assessed by unpaired Student’s T-test or LSD in one-way ANOVA. *p<0.05, **p<0.01, ***p<0.001. [file 40168_2022_1289_MOESM3_ESM.tif]
