# Supplementary material for: Gut dysbiosis promotes prostate cancer progression and docetaxel resistance via activating NF-κB-IL6-STAT3 axis
Source: Microbiome. 2022 Jun 16;10:94. doi: 10.1186/s40168-022-01289-w (PMC9202177; doi:10.1186/s40168-022-01289-w)
Supplement: Supplementary file 5 — Additional file 4: Supplement Table 1. The primer sequences used in the present research. [file 40168_2022_1289_MOESM4_ESM.docx]

**Supplement Table 1 The primer sequences used in the present research**

| Gene |  | Primer sequence (5' to 3') |
| --- | --- | --- |
| IL6 | Forward | 5'-CTGCAAGAGACTTCCATCCAG-3' |
|  | Reverse | 5'-AGTGGTATAGACAGGTCTGTTGG-3' |
| IL1β | Forward | 5'-GAAATGCCACCTTTTGACAGTG-3' |
|  | Reverse | 5'-TGGATGCTCTCATCAGGACAG-3' |
| TNFα | Forward | 5'-CAGGCGGTGCCTATGTCTC-3' |
|  | Reverse | 5'-CGATCACCCCGAAGTTCAGTAG-3' |
| TGFβ | Forward | 5'-CCACCTGCAAGACCATCGAC-3' |
|  | Reverse | 5'-CTGGCGAGCCTTAGTTTGGAC-3' |
| IL10 | Forward | 5'-CTTACTGACTGGCATGAGGATCA-3' |
|  | Reverse | 5'-GCAGCTCTAGGAGCATGTGG-3' |
| VEGF | Forward | 5'-GCACATAGAGAGAATGAGCTTCC-3' |
|  | Reverse | 5'-CTCCGCTCTGAACAAGGCT-3' |
| β-actin | Forward | 5'-GTGACGTTGACATCCGTAAAGA-3' |
|  | Reverse | 5'-GCCGGACTCATCGTACTCC-3' |
