# Supplementary material for: Gut dysbiosis promotes prostate cancer progression and docetaxel resistance via activating NF-κB-IL6-STAT3 axis
Source: Microbiome. 2022 Jun 16;10:94. doi: 10.1186/s40168-022-01289-w (PMC9202177; doi:10.1186/s40168-022-01289-w)
Supplement: Supplementary file 6 — Additional file 5: Supplement Table 2. β-diversity for mice (above) and human (below) of 16S rRNA sequence. [file 40168_2022_1289_MOESM5_ESM.docx]

**Supplement Table 2 β-diversity for mice (above) and human (below) of 16S rRNA sequence**

| groups | Df | Sums of Sqs | Mean Sqs | F-value | R^2^ | *p*-value | significant |
| --- | --- | --- | --- | --- | --- | --- | --- |
| Abx vs FMT-Abx | 1 | 0.5414 | 0.5414 | 10.026 | 0.5562 | 0.008 | ** |
| Abx vs NC | 1 | 0.9147 | 0.9147 | 14.5964 | 0.646 | 0.012 | * |
| FMT-Abx vs FMT-NC | 1 | 0.8216 | 0.8216 | 47.4567 | 0.8557 | 0.011 | * |
| FMT-NC vs NC | 1 | 0.2119 | 0.2119 | 8.1559 | 0.5048 | 0.01 | * |
| Abx vs FMT-Abx vs FMT-NC vs NC | 3 | 2.1293 | 0.7098 | 17.7489 | 0.7689 | 0.001 | ** |
| BPH^a^ vs mPCa^a^ | 1 | 0.1393 | 0.1393 | 1.4375 | 0.0588 | 0.159 | ns |
| BPH vs nmPca^a^ | 1 | 0.151 | 0.151 | 1.345 | 0.0458 | 0.178 | ns |
| nmPCa vs mPCa | 1 | 0.1092 | 0.1092 | 0.9274 | 0.0666 | 0.486 | ns |
| BPH vs nmPCa vs mPCa | 2 | 0.2758 | 0.1379 | 1.2781 | 0.074 | 0.159 | ns |

^a^BPH = benign prostatic hyperplasia; nmPCa = non-metastatic prostate cancer; mPCa = metastatic prostate cancer;

Statistical significance was assessed by Adonis test. **p*<0.05, ***p*<0.01, ****p*<0.001.

Df=degrees of freedom; Sums of Sqs=Sum of squares of deviations; Mean Sqs=Mean squares of deviations
