# Supplementary material for: Gut dysbiosis promotes prostate cancer progression and docetaxel resistance via activating NF-κB-IL6-STAT3 axis
Source: Microbiome. 2022 Jun 16;10:94. doi: 10.1186/s40168-022-01289-w (PMC9202177; doi:10.1186/s40168-022-01289-w)
Supplement: Supplementary file 9 — Additional file 8. Sequencing data for fecal samples of both PCa patients and mice. [file 40168_2022_1289_MOESM8_ESM.docx]

Sequencing data for fecal samples of both PCa patients and mice are in excel files.

Raw data have been deposited in the NCBI Sequence Read Archive (SRA) database and are now available (accession number: PRJNA 792067).

For the concerns about data privacy before publication, we assigned the random numbers to every sample of humans and mice, and the corresponding relation between the samples and numbers are as follows:

**Sample** **number**

**Mouse**:

Abx-1 5

Abx-2 3

Abx-3 11

Abx-4 12

Abx-5 17

FMT-Abx-1 6

FMT-Abx-2 4

FMT-Abx-3 1

FMT-Abx-4 14

FMT-Abx-5 13

FMT-NC-1 20

FMT-NC-2 19

FMT-NC-3 7

FMT-NC-4 2

FMT-NC-5 8

NC-1 9

NC-2 10

NC-3 15

NC-4 16

NC-5 18

**Human**:

BPH-XXZ 17

BPH-ZYG 29

BPH-XDG 21

BPH-CR 25

BPH-JXX 30

BPH-SXC 33

BPH-CZS 22

BPH-CDC 24

BPH-YYD 16

BPH-LWW 19

BPH-WJM 27

BPH-GGQ 20

BPH-LSY 35

BPH-WYT 31

BPH-LZH 28

BPH-CCN 26

BPH-LHH 14

BPH-SJX 9

BPH-BYX 3

BPH-LQH 15

NMPCA-HCS 8

NMPCA-LZD 13

NMPCA-LGL 10

NMPCA-LJX 18

NMPCA-WLS 34

NMPCA-LYJ 12

NMPCA-YPQ 7

NMPCA-WXD 6

NMPCA-LXZ 5

NMPCA-FGL 1

MPCA-YBQ 2

MPCA-LXP 4

MPCA-LRG 11

MPCA-HXL 32

MPCA-ZJX 23
